# Evaluation of Influenza Vaccination Efficacy: A Universal Epidemic Model

**DOI:** 10.1155/2016/5952890

**Published:** 2016-09-07

**Authors:** Lily Ph. Nizolenko, Alexander G. Bachinsky, Sergei I. Bazhan

**Affiliations:** Vector State Research Center of Virology and Biotechnology, Koltsovo, Novosibirsk Region 630559, Russia

## Abstract

By means of a designed epidemic model, we evaluated the influence of seasonal vaccination coverage as well as a potential universal vaccine with differing efficacy on the aftermath of seasonal and pandemic influenza. The results of the modeling enabled us to conclude that, to control a seasonal influenza epidemic with a reproduction coefficient *R*_0_ ≤ 1.5, a 35% vaccination coverage with the current seasonal influenza vaccine formulation is sufficient, provided that other epidemiology measures are regularly implemented. Increasing *R*_0_ level of pandemic strains will obviously require stronger intervention. In addition, seasonal influenza vaccines fail to confer protection against antigenically distinct pandemic influenza strains. Therefore, the necessity of a universal influenza vaccine is clear. The model predicts that a potential universal vaccine will be able to provide sufficient reliable (90%) protection against pandemic influenza only if its efficacy is comparable with the effectiveness of modern vaccines against seasonal influenza strains (70%–80%); given that at least 40% of the population has been vaccinated in advance, ill individuals have been isolated (observed), and a quarantine has been introduced. If other antiepidemic measures are absent, a vaccination coverage of at least 80% is required.

## 1. Introduction

Mathematic approaches can be useful to assess the dynamics of infectious disease epidemics, especially the influence of intervention strategies including large-scale vaccination.

Vaccination is broadly used when controlling epidemics including seasonal influenza, since it provides both direct protection of vaccinees and indirect protection of all individuals due to a decrease in infectious intensity.

Currently used influenza vaccines are efficient if antigenic matching with epidemic strains is desired. Therefore, seasonal vaccines have to be reformulated almost annually. Furthermore, vaccines against seasonal influenza fail to provide protection against pandemic influenza viruses with significantly different antigenic structure. Thus, due to both the permanent threat of the next pandemic and the continual emergence of seasonal influenza A virus variants, there is a need for a universal vaccine providing protective immunity against all or at least the majority of influenza virus variants [1, 2].

In recent years, a number of studies devoted to the design of such vaccines have been published [3–6]. However, researchers usually pay attention only to the immunologic efficacy of their candidate vaccines. Data concerning real prophylactic (epidemiologic) efficacy in the population are actually unavailable before carrying out phase II clinical trials of a potential vaccine. In a smaller number of reports on modeling the influence of such vaccines on influenza epidemic dynamics, this prophylactic efficacy index was either specified on the level of seasonal vaccines or varied slightly [7].

Herein, we designed an epidemic model that enables us to evaluate the influence of vaccination coverage established on the course of seasonal and pandemic influenza epidemic, using existing seasonal and hypothetical universal influenza vaccines with differing efficacies.

## 2. Materials and Methods

At Vector State Research Center of Virology and Biotechnology, we developed a universal deterministic epidemic model according to the population subclasses during the epidemic: S: susceptible persons, E (exposed): infected in incubation period, I: infectious persons, R: recovered, F (fatal): dead persons; hence, the model type was called SEIR(F). In fact, those who died do not form a separate state but are simply removed from the modeling process at a disease-specific death rate. It is assumed that this model is able to simulate the development of any local epidemic of an acute infectious disease, with the infection coming from a certain external source or person-to-person contact independently of sex, age, and other sociodemographic characteristics as the major transmission pathways.

A detailed model description, including equations used, variable parameters, and processes modeled, as well as some data concerning the model's verification was previously published [8]. Initially, the model was adapted to special danger infectious agents, such as smallpox, anthrax, plague (pneumonic and bubonic), tularemia, and hemorrhagic fevers (i.e., Ebola, Marburg, Lassa, and Crimean-Congo). Further, we added socially significant influenza and cholera to the list of modeled infections.

The model is installed on the server at Vector State Research Center of Virology and Biotechnology and is available at http://www.epimod.vector.nsc.ru/ (also available at http://vector-epimod.ru/) and provided with a Web-interface.

The user can edit the model's parameters and divide them into two groups. The first includes parameters characterizing infection per se: infectivity of ill persons and sensitivity to infection, mortality during different stages of a disease, and sensitivity to treatment. The second group includes parameters setting terms and intensity of implementation of interventions: three levels of antiepidemic measures, mainly differing in speed of detection and isolation (supervision) of infected persons, contacts and those suspected of having the disease, and speed of vaccination and quarantine. Moreover, a number of parameters use regional characteristics for which the epidemic is modeled, including its resource provision. It should be noted that evaluation of the most parameter values made according to results of literature data is often arbitrary. In fact, available literature lacks precise qualitative values of one or another index. At best, it is possible to obtain limits, though sometimes only verbal descriptions are available. A number of modeling parameters used in this research are described in Results and Discussion.

Intervention optimization is one of the possibilities of the model used in this study. For this purpose, by specifying initial values and admissible limits of factors by means of multiple automatic simulations, it is possible to obtain values by minimizing a certain criterion:

(1) $$\begin{array}{c} F=\underset{i}{\sum }V_{i}\times f_{i}+\underset{j}{\sum }L_{j}\times g_{j}. \end{array}$$

Maintaining a certain level of epidemic preparedness and using interventions requires specific investments, both material and human. Therefore, the “cost” *f*_*i*_ of an item of each optimization factor *V*_*i*_ is specified. The sum of expenses to maintain or apply specified factor values is included in the optimization criterion as the first component. The second criterion item is the loss caused by the epidemic that depends on the number of its indices (*L*_*j*_, i.e., number of infected persons and number of fatalities, etc.) and user-specified weights of those indices (*g*_*j*_).

A genetic algorithm is used for optimization [9]. Its application in our model has been previously described [10]. We describe optimization parameters in Results and Discussion, since they require an additional explanation.

We considered a megapolis with a population of one million people in our modeling scenario. Initially, 50 infected people at the beginning of the latent stage of disease emerged in the population (i.e., start point). Antiepidemic measures started 20 days after the start point. The time of calculation corresponds to 100 epidemic days.

Model adaptation to different agents included adjustment of modeling parameters corresponding to a certain agent. Adaptation is carried out based on an analysis of data in the literature. In the case of seasonal influenza, we recommend that one should use parameter values listed in Table 1, where necessary explanations are also provided as follows.

(1) For instance, for the latent stage it means that its minimum duration is 4 − 3 = 1.
(2) In the model, population is homogeneous by default; however, for each infected person we defined a group of contacts with a significantly higher probability of infection compared to another population. Thus, the average number of contacts infected by one ill person (*R*_0*c*_) can be considerably higher than the average number of persons infected during “accidental” contacts (*R*_0*s*_). The average number of contacts for one infected person is dependent on the disease.
(3) Since the level of protection provided by administration of prophylactic drugs is similar to the level provided by vaccination [11, 12], it is chemoprophylaxis that substitutes for reactive vaccination when modeling. Furthermore, protective action arrives almost directly after its application. Consequently, duration of establishing postvaccination immunity is set at one day. Accordingly, the values of the model parameters that determine the speed and extent of vaccination, are specified so as to lack resource limits. Unfortunately, the model lacks the possibility to differentiate between chemoprophylaxis and reactive vaccination. Therefore, in the case of influenza, we evaluate only vaccination (preepidemic) efficacy and its influence on the course of the epidemic.
(4) The parameter “rate of infection activity in final stage” specifies the distribution of infection activity between prodromal and final stages of disease. Its meaning is the ratio of the average number of persons becoming infected by one patient in the final stage to an average number of persons becoming infected by one patient throughout the disease (*R*_0_). Here, we assume that disease nosology will have already been determined, doctors will have given recommendations, and familial isolation of infected persons will have been implemented in the final stage.
(5) In practice, infectivity of persons suffering from a mild form of a disease can be considerably lower. However, usually these individuals lead a more active lifestyle and have more contact with a large number of people.
(6) As is known, influenza transmission is limited by close-contact groups (e.g., family, school, and place of work) [13–15]; therefore, infection of contacts is specified separately.
(7) In the case of seasonal influenza, deaths make an inessential contribution to epidemic dynamics. When modeling, they are distributed according to groups of infected persons and disease stages so as to globally provide mortality at the rate of 0.1% in the case of seasonal influenza [16].

When modeling, the influence of immunity on epidemic dynamics is primarily implemented by means of a change in the effective reproduction coefficient *R*_*e*_, determined in the model as an “average number of individuals infected by one ill person.” Related literature often used the reproduction coefficient *R*_0_, denoting the number of individuals infected by one nonimmune ill person in a population of entirely sensitive people under conditions of lacking intervention [17, 18]. Decreases in *R*_*e*_ values compared to *R*_0_ are reached, for example, by influence of the level of vaccination coverage. Primarily, immune people can be less sensitive to infection. Secondly, their infectivity can be reduced. Thirdly, the proportion of a severe form of the disease (if any) can be lower than in nonimmune persons.

Usually, vaccine efficacy represents a reduction of morbidity in vaccinees compared to unvaccinated persons [19–22]. Some models describe the impact of vaccination on the probability of developing severe forms of the disease [23]. The difference in the infectious activity of mild and severe forms is also taken into account in several models [19, 24]. However, models using all indicators simultaneously are unknown.

Our model specificity includes a broader range of parameters differentiating immune people from those who are not immune. Therefore, in the frame of the model vaccine, prophylactic efficacy can be evaluated according to its influence on values of *S*, *Q*, and *H* parameters (see explanations after (2)). Consequently, if *P* is the rate of immune people in the population, then the dependence of *R*_*e*_ on that value can be formulated as follows:

(2) Re=P×S+1−P×R0s×P×Q×H+1−Q×E+1−P×q+1−qE+R0c,

where *P* is rate of immune people in the population, *S* ≤ 1 is coefficient specifying proportion of sensitivity to infection in immune people in relation to sensitivity of nonimmune persons, *q* is rate of a severe forms among nonimmune people, *Q* is rate of a severe forms among immune people (*Q* ≤ *q*), *E* ≤ 1 is the rate between transmissibility of ill person with the mild form of a disease and transmissibility of people with severe disease form, *H* ≤ 1 is the rate of transmissibility of immune people with the severe disease form to transmissibility of nonimmune people with severe disease form (transmissibility in nonimmune people with severe disease = 1), *R*_0*s*_ is average number of people infected by one ill person in entirely sensitive population under random contacts, and *R*_0*c*_ is average number of people infected by one ill person in entirely sensitive population among close contacts.

Expression in the first square brackets specifies average reduction of sensitivity to infection in the population by means of the presence of immune people. Expression in the second square brackets specifies reduction of intensity of infection background (formed by ill people) that can also depend on the level of immunity, partially due to different occurrence probability of severe and mild forms of disease among immune and nonimmune people.

In the case of seasonal influenza, the model defaults to using the values for those parameters, *R*_0_ = 1.5, *S* = 0.2; *Q* = 0.2; *q* = 0.5; *E* = 0.5; and *H* = 0.5, selected based on analysis of data in the literature including those regarding effectiveness of currently used vaccines [11, 25–28].

When modeling influenza epidemic dynamics, results of other research groups correspond to actual data where the correlation between *R*_0*s*_ and *R*_0*c*_ is approximately equal to 1 : 2 [14, 15, 29]. This correlation was also used in our model. For example, as shown in Table 1, the mean number of contacts per infected person is 5, rate of infected contacts per day is 4%, and the average duration of infectiousness is 5 days. As a result, the average number of people infected by one ill person among close contacts (*R*_0*c*_) is equal to 1. Then, the value of *R*_0*s*_ is 0.5.

## 3. Results and Discussion

### 3.1. Model Validation

The above arguments are not conventional, but they are consistent with the known methods. According to the simple well-known formulas for the critical vaccination coverage for herd immunity, the proportion of immune people should exceed

(3) $$\begin{array}{c} P\geq 1-\frac{1}{R_{0}}, \end{array}$$

assuming 100% vaccine efficacy, or

(4) $$\begin{array}{c} P\geq \frac{1-1/R_{0}}{Vaccine\;efficacy}, \end{array}$$

in the case when vaccination efficacy is not 100% [22, 36]. For *R*_0_ = 1.5, incidence of infection would decline if the proportion of immune persons exceeded 33%–41% (100% and 80% vaccine efficacy, resp.). According to formula (2), *R*_*e*_ reduces with increasing *P*, and at a certain level of vaccination coverage *R*_*e*_ can be <1. Therefore, the epidemic lessens, even in the absence of other interventions. This is observed for seasonal influenza with *R*_0_ = 1.5 in the absence of other interventions but in the presence of a ≥35% vaccinated population (*R*_*e*_ < 1).

Results of the modeling in the presence of a 35% immunized population show that an epidemic actually ends naturally without other antiepidemic measures (including both isolation of ill persons and observation of the contacts) and quarantine. However, its duration is significantly long (about a year). By the 100th day of calculation, more than 19,000 people are infected and 30 fatal cases are registered (maximum mortality rate, equal to 0.2% (severe form) among infected people). Provided that the full range of antiepidemic measures is undertaken, only 637 people are infected, with two cases leading to mortality. In the case of a 41% immunized population, the indices are equal to 4,527 and 11 without other antiepidemic measures and 476 with one fatal case when ill individuals have been isolated (observed) and quarantine has been introduced.

Thus, the simulation results show that the provision of vaccination coverage at the level determined by formulas (3) and (4), as well as formula (2), is a necessary but not sufficient condition for successful flu epidemic control.

This conclusion is confirmed by the real epidemic. For example, according to the Research Institute of Influenza (Ministry of Health of the Russian Federation) [37], a large outbreak of influenza H1N1 was observed in St. Petersburg at the beginning of 2016. It was fixed within 3–8 weeks of the year with a peak in the fifth week, when it was observed to exceed the Influenza-Like Illness (ILI) morbidity epidemic thresholds for the overall population by 129%. This week, about 97,000 people fell ill. Moreover, as stated in the Resolution of the Chief Medical Officer of St. Petersburg [38], the relative share of influenza among other acute respiratory infections was 60%. Despite the fact that about 50% of the population of St. Petersburg has been previously vaccinated, the epidemic was rapidly developing until a full range of antiepidemic measures was launched at the end of the fourth week of 2016.

**Figure 1:**
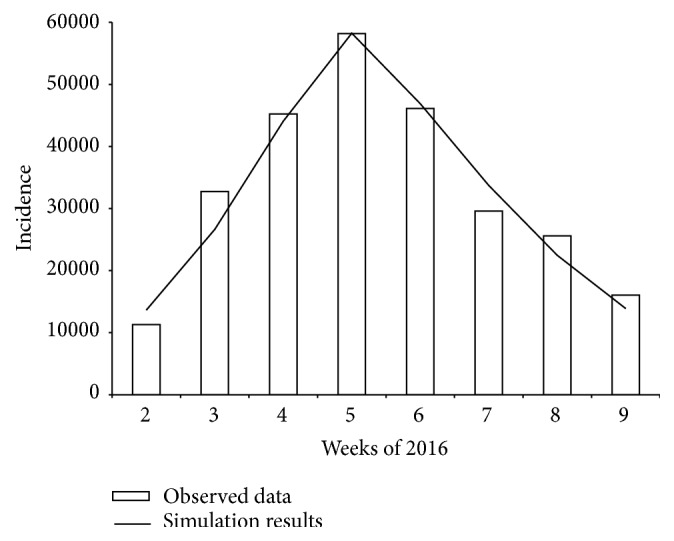
Observed data and simulation results for the 2016 influenza epidemic in St. Petersburg.

The simulation results showed dynamics very similar to the epidemic at *R*_0_ = 1.6, with a rate of immune persons at 50% and the implementation of other control activities starting from 4 weeks (Figure 1). Note that, theoretically, vaccination coverage in St. Petersburg was sufficient to control the epidemic. For *R*_0_ = 1.6, critical vaccination coverage is 41% when calculated according to formula (2) and 38%–47% by formulas (3) and (4).

### 3.2. Vaccination Coverage during Seasonal Influenza Epidemic Required for Controlling Disease Transmission

To specify the role of vaccination among other measures, we conducted an optimization of interventions.  Table 2 demonstrates the limits of factors for optimization and the “cost” of unity for each factor.

The goal of our research was to evaluate specific influence of vaccination on epidemic aftermath; therefore, the value of the upper limit of the parameter “rate of immune persons” is specified at a high level (at least 50%) and for other parameters is specified within the limits of model city resources. Since the genetic algorithm used for optimization is stochastic, we carried out a fivefold optimization for each set of parameters.

Calculations suggest that despite the intentionally overestimated “cost” of the unity of “vaccination coverage” (5–10-fold higher compared to the most “expensive” other parameters, such as expenses for bed capacity and medical staff) in optimized conditions, this parameter always takes the maximum permitted values (equal to an upper limit of 50% or 60%). When we specified a 70% upper limit of the rate of immune people, the maximum value of that parameter in optimized conditions reached 65%-66%. Besides, the higher the limit of the number of immune people, the lower the influence of all other parameters. At the upper level of the number of immune people (50%–60%), values of parameters describing the intensity of antiepidemic measures (i.e., a rate of daily isolated nonimmune ill persons) in optimized conditions were similar or reached the upper limit. All other parameters had values as low as the minimum permissible. A sample of parameter values in optimized conditions at the upper limit of the rate of immune people is 60%, observed in the last column of Table 2.

At a vaccination coverage level of 65% or higher, all other parameters had minimum permissible (zero) values. In other words, any antiepidemic measures were unnecessary. At the same time, “cost” of the epidemic was significantly reduced (Table 3).

Table 3 shows that, under otherwise equal conditions, the increase in rate of immune people among the population from 20% (before optimization) to 50% results in a fivefold reduction of the number of infected people. Further increase of the level of vaccination coverage does not lead to the same significant effect, and lowering the cost of an epidemic is primarily accounted for by reduction or full absence of expenses necessary for maintenance of high levels of antiepidemic protection.

### 3.3. Immunization against Pandemic Strains

Here, we estimate the influence of effectiveness of a potential universal vaccine on pandemic influenza as long as it is used before the epidemic in the same model city. We did not study the case of vaccination just in the course of the epidemic, as well as the vaccination/revaccination regimens themselves. It was believed that by the time of calculations, immunity of vaccinees had to be entirely established.

When modeling, pandemic variants differed from seasonal ones by an enhanced reproduction coefficient (*R*_0_  1.5 → 2.5), increased mortality rate (five-fold), and low level of natural herd immunity [16, 39, 40]. When modeling, we specified that total herd immunity is formed before the beginning of calculations due to vaccination.

Since characteristics of a potential universal vaccine are previously unknown, the change of “vaccine efficiency” stands for scaling all modeling parameters that are different in immune and nonimmune people. For example, if vaccine efficacy is equal to 90%, then coefficients specifying the rate of sensitivity to infection in immune people as related to sensitivity in nonimmune people (*S*) and relation of transmissibility of immune people to transmissibility in nonimmune people (*H*) were specified at the level of 0.1, and the rate of severe courses among immune people (*Q*) was 0.04 compared to the rate of severe courses among nonimmune people (*q* = 0.4). Moreover, the mortality rate of immune people decreases 10-fold compared to nonimmune individuals at any stage of disease. Of note, according to calculations, proportion/disproportion has no impact on epidemic dynamics.

In theory, the influence of vaccine efficacy and the level of its usage can be evaluated by detection of the effective rate of transmission *R*_*e*_, as it is carried out for seasonal influenza (formula (2)).  Table 4 shows values of parameter *R*_*e*_ for different combinations of vaccine efficacy and vaccination coverage. In such conditions, a potential universal vaccine of the same level of efficacy as modern seasonal vaccines (i.e., approximately 80%) can provide protection against pandemic influenza when vaccination coverage reaches at least 80% of the population. This result is consistent with work of others [22]; however, as previously demonstrated, it is insufficient to control the actual epidemic.

For a good understanding of the level of the influence of one parameter or another on epidemic aftermath, it makes sense to introduce a concept of population protectability. The following value is considered the protectability index:

(5) $$\begin{array}{c} P_{i}=\frac{N_{0}-N_{i}}{N_{0}}, \end{array}$$

where *N*_0_ is a value of a certain index (in this context, number of infected people) by the finish of calculation in the absence of analyzed intervention and *N*_*i*_ is the value of the same index in the case of implementation of intervention “*i*.”

**Figure 2:**
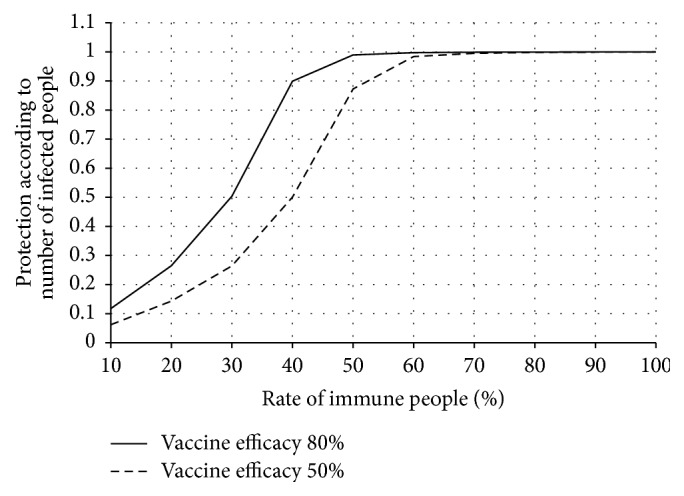
Comparison of protection according to the number of infected persons for different levels of vaccination coverage established before the beginning of pandemic influenza using potential vaccines of differing efficacy.

Figure 2 compares protectability indices for different levels of vaccination coverage established by the use of vaccines with 50% and 80% efficacy.

Influenza epidemic dynamics were being calculated for 100 days since registering the first infected people at different levels of vaccination coverage.

Modeling results enable us to conclude that a potential universal vaccine of the same level of efficacy as modern seasonal vaccines (i.e., approximately 80%) can provide sufficiently reliable (90%) protection against pandemic influenza under conditions of previous vaccination of at least 40% of the population. We emphasize vaccination since natural immunity in the population is believed to be extremely low. Furthermore, when computing this parameter, the full range of antiepidemic measures is carried out during a seasonal influenza epidemic (partial isolation of ill people, quarantine, and prophylactic treatment). At 50% efficacy of a universal vaccine, at least 50% of the population should be immunized to reach the same level (90%) of protection.

## 4. Conclusions

The tool, developed in the SRC VB Vector, is based on the model simulating epidemics of acute infectious diseases, where the main modes of transmission are from an external source or by casual contact between people. Unlike most models available on the Internet, this tool is designed for use by epidemiologists and policy makers who not always own the means of information technologies. The user has access to all parameters of the model. Guided by extensive instructions on how to work with the model, as well as detailed descriptions of infections, the user can specify particular infections and the population, where the epidemics are simulated and play various scenarios simulating epidemics taking into account the presence or absence of various resource constraints.

This simulation and optimization of intervention can not only help to understand the impact of various factors on the development of the epidemic but also help to develop the best strategy to counter reduce losses from epidemics, as well as determine the resources required and sufficient for a successful fight against epidemics.

Modeling results enable us to conclude that, to control seasonal influenza epidemic at *R*_0_ equal to 1.5, it is sufficient to maintain a vaccination coverage level of 35%, but only under the condition that other antiepidemic measures are routinely carried out.

Increasing *R*_0_ level in the case of emergence of a pandemic influenza virus strain will obviously require implementation of stronger interventions. The model predicts that, in the case of efficacy of a potential universal vaccine that is comparable to the efficacy of modern vaccines against seasonal influenza strains (i.e., approximately 80%), it can provide sufficiently reliable (90%) protection against pandemic influenza; given that at least 40% of the population has been previously immunized, ill persons had been isolated (observed), and quarantine had been introduced. In the absence of other antiepidemic measures, vaccination coverage of at least 80% is required.

## Figures and Tables

**Table 1:** Parameters used for calculations of seasonal influenza outbreak dynamics.

Parameter	Value	Note
Maximum duration of each stage during disease development (latent, prodromal, and final), days	4; 3; 4	[30–32]
Day when disease passes from one stage to the other (see (1))	3; 2; 2	[32, 33]
Mean number of contacts per one infected person	5	See (2)
Duration of establishing postvaccination immunity, days	1	See (3)
Rate of infectious activity in final stage, %	20	See (4)
Rate of infectious activity of immune to nonimmune patients, %	50	[11]
Rate of infectious activity of cases with mild to severe forms, %	10	See (5)
Rate of severe forms among nonimmune patients, %	40	[34]
Rate of severe forms among immune patients, %	20	[35]
Rate of sensitivity to infection of immune patients relative to nonimmune ones, %	20	[25, 26]
Rate of infected contacts per day, %	4	See (6)
Mortality rate of nonimmune patients in prodromal stage, %	0.1	See (7)
Mortality rate of immune patients in prodromal stage, %	0.03	See (7)
Mortality rate of nonimmune patients with severe form in final stage, %	0.2	See (7)
Mortality rate of immune patients with severe form in final stage, %	0.05	See (7)
Mortality rate of patients with mild form in final stage, %	0.01	See (7)
Decrease in mortality rate of treated cases, %	30	[12]
Duration of contacts observation, days	4	[30–32]

**Table 2:** Limits and nominal “costs” of optimization indices when modeling seasonal influenza epidemic.

Parameter	Parameter limits	The unit “cost”	Parameter value in optimized conditions at the upper limit of rate of immune people (60%)^1^
Rate of immune persons, %	0–50 (60; 70)	1000	60

*Parameters of antiepidemic measures*			
Rate of daily isolated asymptomatic contacts/suspects, %	0–10	10	0–2
Rate of daily isolated patients in prodromal stage (nonimmune), %	0–10	10	0–8
Rate of daily isolated patients in prodromal stage (immune), %	0–8	10	0-1
Rate of daily isolated patients in final stage (severe case), %	0–15	10	0–3
Minimal rate of daily isolated symptomatic patients, %	0–2	10	0–2
Maximal rate of people having started daily to obtain prophylactic treatment in risk groups, %	0–50	5	0–12

*Parameters of resources*			
Number of medics/paramedics involved in epidemic eradication	100–2000	100	100
Number of teams searching for and isolating or observing infected cases and contacts	0–100	200	1
Number of patients/contacts detected by one team per day	0–20	50	5
Number of units issuing chemoprophylactic items	0–500	100	0
Number of people daily serviced in one unit	0–500	50	0
Stock of prophylactic items	0–1000000	2	0
Reserve of drugs (for one treatment course)	0–1000000	3	0
Bed capacity for strict isolation	0–30000	100	0–2
Bed capacity in provisional hospitals	0–2500	50	0
Bed capacity in quarantine departments for contacts	0–10000	70	0

^1^Minimal and maximal value for optimization parameters obtained after five iterations of the procedure.

**Table 3:** Comparison of aftermath of seasonal influenza epidemic under optimized conditions at different vaccination coverage.

Parameters	Nominal “cost” of parameter	Before optimization	In optimized conditions at the upper limit of rate of immune people		
			50%	60%	70%
Total number of infected persons	100	1879	361–439	199–214	150–159
Total number of lethal cases	1000000	6	1	1	0
Person days of isolated patients	1	1410	23–161	0–22	0–6
Person days of observed contacts	0.1	5041	0–521	0	0
Epidemic “cost”: expenses + losses in nominal units		6014424	202259–237887	190582–191680	91074–91276

**Table 4:** Value *R*_*e*_ for parameters combination: level of vaccination coverage versus vaccine efficacy against pandemic influenza at *R*_0_ = 2.5. Bold numerals highlight combinations of parameters preventing epidemic from significant spreading (*R*_*e*_ < 1) in the absence of other interventions.

Rate of immune persons, %	Vaccine efficacy, %					
	100	90	80	70	60	50
0	2.5	2.5	2.5	2.5	2.5	2.5
10	2.2	2.2	2.1	2.3	2.3	2.3
20	2.0	2.0	1.9	2.1	2.1	2.2
30	1.7	1.8	1.6	1.9	2.0	2.1
40	1.4	1.5	1.4	1.7	1.8	1.9
50	1.2	1.3	1.2	1.5	1.7	1.8
60	**0.9**	1.1	1.1	1.4	1.5	1.7
70	**0.7**	**0.9**	**0.9**	1.2	1.4	1.5
80	**0.5**	**0.6**	**0.7**	**1.0**	1.2	1.4
90	**0.2**	**0.4**	**0.5**	**0.8**	1.1	1.3
100	**0.0**	**0.2**	**0.4**	**0.6**	**0.9**	1.2

## References

[1] Zhang H., Wang L., Compans R. W., Wang B.-Z. (2014). Universal influenza vaccines, a dream to be realized soon. *Viruses*.

[2] De Vries R. D., Altenburg A. F., Rimmelzwaan G. F. (2015). Universal influenza vaccines, science fiction or soon reality?. *Expert Review of Vaccines*.

[3] Fiers W., De Filette M., Birkett A., Neirynck S., Min Jou W. (2004). A ‘universal’ human influenza A vaccine. *Virus Research*.

[4] De Filette M., Fiers W., Martens W. (2006). Improved design and intranasal delivery of an M2e-based human influenza A vaccine. *Vaccine*.

[5] Pica N., Palese P. (2013). Toward a universal influenza virus vaccine: prospects and challenges. *Annual Review of Medicine*.

[6] Soema P. C., Van Riet E., Kersten G., Amorij J.-P. (2015). Development of cross-protective influenza A vaccines based on cellular responses. *Frontiers in Immunology*.

[7] Lee B. Y., Tai J. H. Y., McGlone S. M. (2012). The potential economic value of a 'universal' (multi-year) influenza vaccine. *Influenza and other Respiratory Viruses*.

[8] Bachinsky A. G., Nizolenko L. P. (2013). A universal model for predicting dynamics of the epidemics caused by special pathogens. *BioMed Research International*.

[9] Haupt R. L., Haupt S. E. (2004). *Practical Genetic Algorithms*.

[10] Bachinsky A. G., Nizolenko L. P. (2014). A universal model of epidemic: optimizing interventions. *Universal Journal of Public Health*.

[11] Longini I. M., Halloran M. E., Nizam A., Yang Y. (2004). Containing pandemic influenza with antiviral agents. *American Journal of Epidemiology*.

[12] Regoes R. R., Bonhoeffer S. (2006). Emergence of drug-resistant influenza virus: population dynamical considerations. *Science*.

[13] Addy C. L., Longini I. M., Haber M. (1991). A generalized stochastic model for the analysis of infectious disease final size data. *Biometrics*.

[14] Fraser C. (2007). Estimating individual and household reproduction numbers in an emerging epidemic. *PLoS ONE*.

[15] Fraser C., Cummings D. A. T., Klinkenberg D., Burke D. S., Ferguson N. M. (2011). Influenza transmission in households during the 1918 pandemic. *American Journal of Epidemiology*.

[16] Viboud C., Tam T., Fleming D., Handel A., Miller M. A., Simonsen L. (2006). Transmissibility and mortality impact of epidemic and pandemic influenza, with emphasis on the unusually deadly 1951 epidemic. *Vaccine*.

[17] Diekmann O., Heesterbeek J. A., Metz J. A. (1990). On the definition and the computation of the basic reproduction ratio *R*
_o_ in models for infectious diseases in heterogeneous populations. *Journal of Mathematical Biology*.

[18] Heesterbeek J. A. P. (2002). A brief history of *R*
_0_ and a recipe for its calculation. *Acta Biotheoretica*.

[19] Carrat F., Luong J., Lao H., Sallé A.-V., Lajaunie C., Wackernagel H. (2006). A ‘small-world-like’ model for comparing interventions aimed at preventing and controlling influenza pandemics. *BMC Medicine*.

[20] Lee B. Y., Brown S. T., Cooley P. (2010). Vaccination deep into a pandemic wave: potential mechanisms for a ‘third wave’ and the impact of vaccination. *American Journal of Preventive Medicine*.

[21] Liu J., Xia S. (2011). Toward effective vaccine deployment: a systematic study. *Journal of Medical Systems*.

[22] Plans-Rubió P. (2012). The vaccination coverage required to establish herd immunity against influenza viruses. *Preventive Medicine*.

[23] Sharomi O., Podder C. N., Gumel A. B., Mahmud S., Rubinstein E. (2011). Modelling the transmission dynamics and control of the novel 2009 swine influenza (H1N1) pandemic. *Bulletin of Mathematical Biology*.

[24] Milne G., Kelso J., Kelly H. (2010). Strategies for mitigating an influenza pandemic with pre-pandemic H5N1 vaccines. *Journal of the Royal Society Interface*.

[25] Skowronski D. M., Janjua N. Z., De Serres G. (2011). Effectiveness of AS03 adjuvanted pandemic H1N1 vaccine: case-control evaluation based on sentinel surveillance system in Canada, autumn 2009. *British Medical Journal*.

[26] Valenciano M., Kissling E., Cohen J.-M. (2011). Estimates of pandemic influenza vaccine effectiveness in europe, 2009-2010: results of influenza monitoring vaccine effectiveness in Europe (I-MOVE) Multicentre Case-Control Study. *PLoS Medicine*.

[27] Wallinga J., Lipsitch M. (2007). How generation intervals shape the relationship between growth rates and reproductive numbers. *Proceedings of the Royal Society B: Biological Sciences*.

[28] Chowell G., Miller M. A., Viboud C. (2008). Seasonal influenza in the United States, France, and Australia: transmission and prospects for control. *Epidemiology and Infection*.

[29] Ferguson N. M., Cummings D. A. T., Fraser C., Cajka J. C., Cooley P. C., Burke D. S. (2006). Strategies for mitigating an influenza pandemic. *Nature*.

[30] Bell D. M., Nicoll A., Fukuda K. (2006). Nonpharmaceutical interventions for pandemic influenza, international measures. *Emerging Infectious Diseases*.

[31] Tuite A. R., Greer A. L., Whelan M. (2010). Estimated epidemiologic parameters and morbidity associated with pandemic H1N1 influenza. *Canadian Medical Association Journal*.

[32] Carrat F., Vergu E., Ferguson N. M. (2008). Time lines of infection and disease in human influenza: a review of volunteer challenge studies. *American Journal of Epidemiology*.

[33] Hirotsu N., Ikematsu H., Iwaki N. (2004). Effects of antiviral drugs on viral detection in influenza patients and on the sequential infection to their family members—serial examination by rapid diagnosis (Capilia) and virus culture. *International Congress Series*.

[34] Kuster S. P., Shah P. S., Coleman B. L. (2011). Incidence of influenza in healthy adults and healthcare workers: A systematic review and meta-analysis. *PLoS ONE*.

[35] Wood S. C., Alexseiv A., Nguyen V. H. (1999). Effectiveness and economical impact of vaccination against influenza among a working population in Moscow. *Vaccine*.

[36] Fine P., Eames K., Heymann D. L. (2011). ‘Herd immunity’: a rough guide. *Clinical Infectious Diseases*.

[37] Influenza Surveillance System in Russia: Epidemic situation. http://www.influenza.spb.ru/en/influenza_surveillance_system_in_russia/epidemic_situation.

[38] Resolution of the Chief Medical Officer of St. Petersburg. http://78.rospotrebnadzor.ru/c/document_library/get_file?uuid=4c2e5fd8-71ec-4122-a2a9-94aaa2939d59&groupId=935484.

[39] Cheng K. F., Leung P. C. (2007). What happened in China during the 1918 influenza pandemic?. *International Journal of Infectious Diseases*.

[40] Chowell G., Ammon C. E., Hengartner N. W., Hyman J. M. (2006). Transmission dynamics of the great influenza pandemic of 1918 in Geneva, Switzerland: assessing the effects of hypothetical interventions. *Journal of Theoretical Biology*.

